# Prognostic significance and potential association between ALDOA and ENO1 in gastric cancer

**DOI:** 10.7150/jca.114369

**Published:** 2025-08-22

**Authors:** Xuchao Wang, Yibin Lu, Zhengwu Cheng, Yizhou Yao, Xinyu Shao

**Affiliations:** 1 Department of Gastroenterology, The Affiliated Suzhou Hospital of Nanjing Medical University, Suzhou Municipal Hospital, Gusu School, Nanjing Medical University, Suzhou, China.; 2 Department of General Surgery, The First Affiliated Hospital of Soochow University, Suzhou, Jiangsu, China.; 3 Department of Surgery, Suzhou Industrial Park Xingpu Hospital, Suzhou, Jiangsu, China.; 4 Department of Gastrointestinal Surgery, The First Affiliated Hospital of Wannan Medical College, Wuhu, China.

**Keywords:** Gastric cancer, ALDOA, ENO1, prognosis, glycolysis

## Abstract

**Background:** Gastric cancer (GC) is one of the most common malignant tumors and a leading cause of cancer-related death worldwide. Although advances in surgical techniques and novel treatment techniques such as immunotherapy have improved the prognosis of many tumors, the effectiveness of treatment for advanced GC patients is still limited.

**Methods:** Immunohistochemistry (IHC) staining analysis was conducted to compare the expression of ALDOA and ENO1 in GC tissues and adjacent normal tissues, complemented by bioinformatics analysis using GEPIA, LinkedOmics, and TIMER databases to explore their association with glycolysis and immune cell infiltration. A survival prediction nomogram was constructed based on Cox proportional hazard model data to evaluate prognostic significance.

**Results:** In this study, through IHC staining analysis, it was observed that the expression levels of ALDOA and ENO1 in GC tissues were significantly higher than those in adjacent normal tissues. Moreover, the aberrant expression of ALDOA/ENO1 was associated with a poor prognosis in GC patients. Bioinformatics analysis revealed a positive correlation between ALDOA and ENO1 expression, both intricately associated with glycolysis pathway activation. A survival prediction nomogram, constructed based on the univariate analysis of data from the Cox proportional hazard model, demonstrated that the expression of ALDOA and ENO1 significantly impacts the prognosis of GC patients.

**Conclusions:** ALDOA/ENO1 may play a crucial role in GC, which may potentially offer new perspectives and directions for the development of targeted therapies specifically designed for GC patients.

## Introduction

Gastric cancer (GC) ranks among the most prevalent malignancies and is the fourth leading cause of cancer-related deaths worldwide 1, 2. Due to the limited availability of early endoscopic screening and the lack of reliable tumor markers, many patients are diagnosed at an advanced stage or with drug resistance, which significantly reduces their chances of survival 3-6. Therefore, identifying effective diagnostic and therapeutic targets and elucidating their underlying mechanisms are essential for advancing the treatment of gastric cancer.

ALDOA encodes a glycolytic enzyme that turns fructose-1,6-bisphosphate into glyceraldehyde 3-phosphate and dihydroxyacetone phosphate 7, 8. The proliferation and migration of cancer cells rely heavily on metabolic reprogramming 9, 10. Research has demonstrated that abnormal ALDOA expression is linked to tumor progression due to its role in glycolysis 8, 11, 12. However, changes in ALDOA expression in gastric cancer (GC) and their impact on tumor cells have been scarcely investigated. Furthermore, the precise mechanisms by which ALDOA regulates glycolysis in GC cells remain poorly understood.

In this study, we examined alterations in ALDOA expression and their effect on clinical survival by integrating bioinformatics analysis with the examination of tumor tissues. Additionally, we explored its correlation with ENO1 to uncover potential new therapeutic strategies for GC.

## Materials and Methods

### Collection of human GC tissues

115 GC tissues along with their paired normal tissues were procured from GC patients. These patients had undergone radical gastrectomy during the period from 2015 to 2016 at the First Affiliated Hospital of Wannan Medical College. The inclusion criteria: (1) Patients who underwent radical gastrectomy for primary GC; (2) Histopathologically confirmed GC with paired adjacent normal tissues; (3) Complete clinical data and follow-up (≥5 years). The exclusion criteria: (1) Preoperative chemotherapy/radiotherapy/immunotherapy; (2) Concurrent malignancies or autoimmune diseases; (3) Severe cardiopulmonary function diseases; (4) Severe hepatic/renal dysfunction or metabolic disorders; (5) Incomplete clinical records or follow-up.

The surgical specimens were preserved in 10% formalin and then embedded in paraffin. All patients furnished comprehensive clinical data and were available for follow-up. Written informed consent was obtained from the participating patients. This research adhered to the ethical guidelines of the Helsinki Declaration and received approval from the ethics committees of the Affiliated Suzhou Hospital of Nanjing Medical University (2020304) and the First Affiliated Hospital of Wannan Medical College (202248).

### Bioinformatic analysis

Bioinformatic analysis was carried out using three platform databases, namely the GEPIA database, LinkedOmics database, and TIMER database. The LinkedOmics database (https://www.linkedomics.org/login.php) and GEPIA database (http://gepia.cancer-pku.cn/index.html) were employed to assess the correlation between ALDOA expression and the expression of relevant markers. Additionally, the TIMER database was utilized to analyze immune cell infiltration in GC (https://cistrome.shinyapps.io/timer/). This database was used to evaluate the relationships between the expression of ALDOA and the infiltration levels of immune cell subsets.

### Immunohistochemistry

The collected GC tissues and paired normal tissues were fixed with formalin, embedded in paraffin, sliced into 5-μm sections, and then subjected to immunohistochemical staining. The sections were incubated with anti-ALDOA or anti-ENO1 antibodies at a 1:100 dilution at room temperature for 2 hours. The results were visualized using a tissue staining kit (manufactured by Zhongshan Biotechnology, China). The staining scores were evaluated by two researchers who were blinded to the sample details. Five regions were randomly chosen for staining evaluation. The IHC score was determined by multiplying the intensity score (0 for negative, 1 for weak, 2 for moderate, 3 for strong) and the extent score (0 for 0-5%, 1 for 6-25%, 2 for 26-50%, 3 for 51-75%, 4 for >75%). A final average score ranging from 0 to 4 was regarded as negative, while a final average score of 5 to 12 was considered positive 13.

### Statistical analysis

All procedures were carried out following the pertinent guidelines and regulations. The data are expressed as means ± standard deviation. Statistical evaluations were conducted with SPSS 22.0 (SPSS Inc., Chicago, IL, USA), GraphPad Prism 8 (San Diego, CA, USA), and R (version 3.6.1 for Windows, available at http://cran.r-project.org/). To compare the means between groups, either the t-test (unpaired, two-tailed) or the Mann-Whitney U test was used. A P-value less than 0.05 was deemed to signify statistical significance.

## Results

### Aberrantly increased ALDOA in GC indicates a poor prognosis

**Figure 1:**
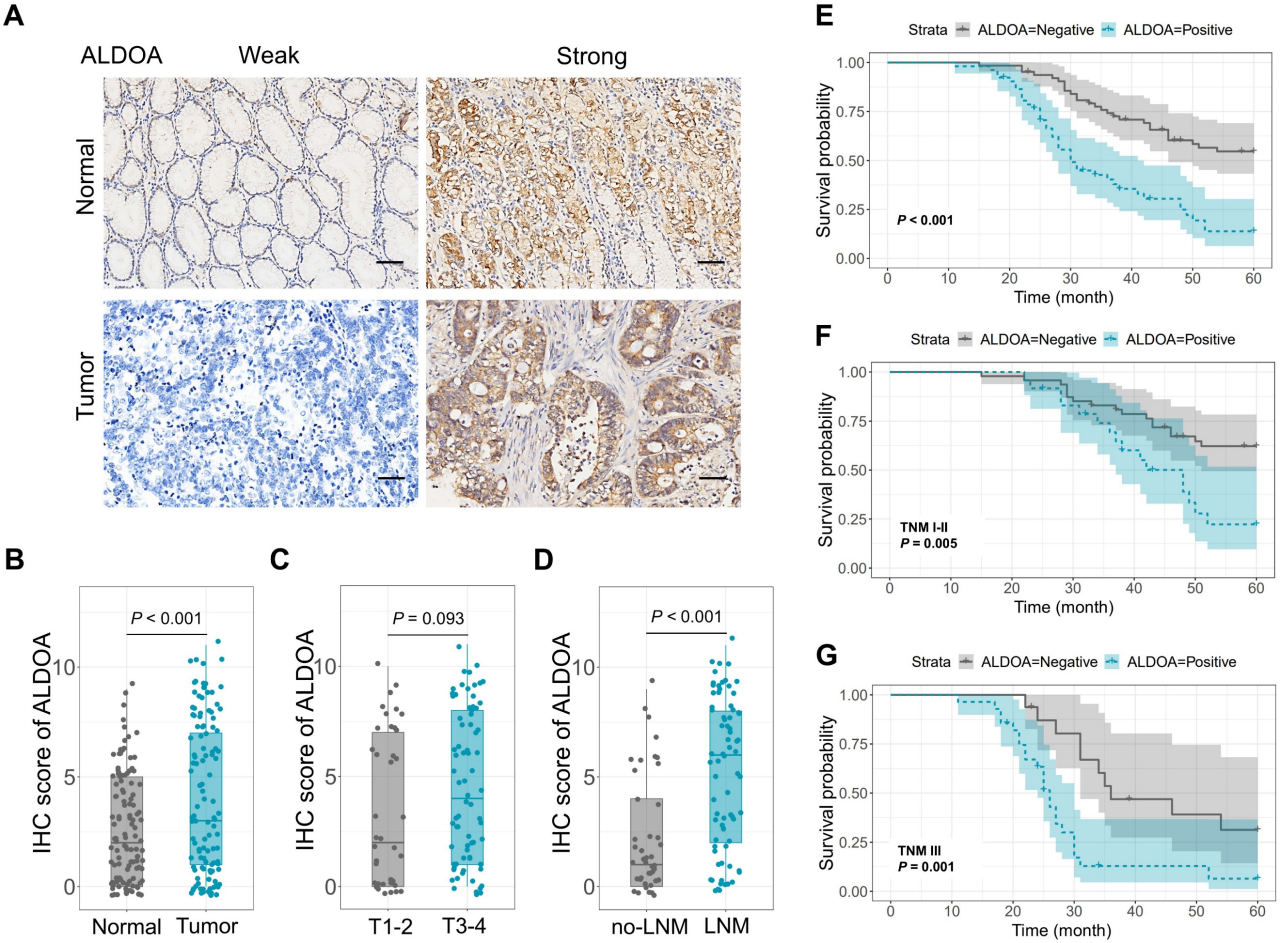
ALDOA expression increased in GC tissues and indicated a poor prognosis. (A) ALDOA expression detected by IHC in 115 GC tissues and paired normal tissues. (B-D) IHC score of ALDOA in (B) GC tissues and paired normal tissues, (C) GC tissues with T1-2 or T3-4, and (D) GC tissues with or without lymph node metastasis. (E-G) Survival analysis of ALDOA expression level in (E) GC patients, (F) GC patients with TNM stage I-II, and (G) GC patients with TNM stage III. Scale bar = 100 μm.

We conducted an immunohistochemistry (IHC) analysis on 115 gastric cancer (GC) tissues and their corresponding normal tissues (Fig. 1A). The results revealed a significant upregulation of ALDOA expression in GC tissues compared to normal tissues (Fig. 1B). When we performed a subgroup analysis comparing T1-2 stage tumors to T3-4 stage tumors, no significant difference in ALDOA expression was observed between the two groups (Fig. 1C). In contrast, in the subgroup analysis distinguishing GC patients with lymph node metastasis from those without, the IHC score indicated a markedly higher expression of ALDOA in the lymph node metastasis group compared to the non-lymph node metastasis group (Fig. 1D).

We also explored the impact of ALDOA expression on the prognosis of GC patients. The patients were then categorized into two groups: ALDOA-positive (ALDOA^pos^) and ALDOA-negative (ALDOA^neg^). It was observed that patients with ALDOA^pos^ had significantly poorer overall survival compared to those with ALDOA^neg^ (P < 0.001, Fig. 1E). Subsequent subgroup analysis revealed that this trend held true for both the TNM stage I-II subgroup (P = 0.005, Fig. 1F) and the TNM stage III subgroup (P = 0.001, Fig. 1G), indicating that GC patients with ALDOA^pos^ expression consistently exhibited worse survival outcomes compared to those with ALDOA^neg^.

### ALDOA is related to ENO1-dependent glycolysis

**Figure 2:**
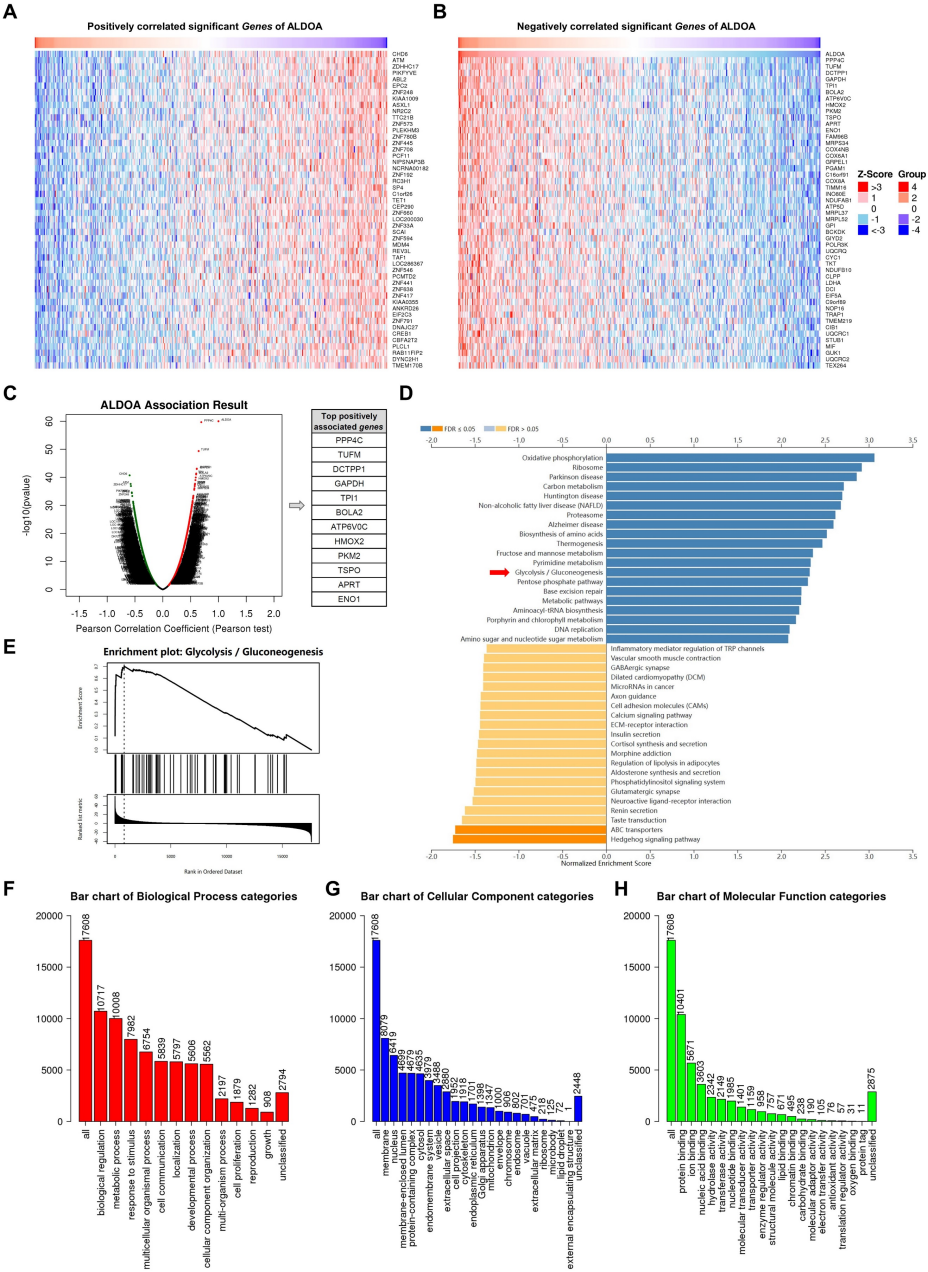
Enrichment analysis of ALDOA functions in GC. (A-B) The top genes positively (A) and negatively (B) associated with ALDOA in GC are displayed. (C) Volcano plots displaying ALDOA-related genes in GC. (D) KEGG analysis indicating that ALDOA is involved in various pathways. (E) GSEA of glycolysis gene sets. (F-H) GO enrichment analysis showing correlations with (F) biological processes, (G) cellular components, and (H) molecular functions.

In this study, ALDOA has been identified as a promising diagnostic and prognostic marker for gastric cancer (GC) patients, although the underlying mechanisms are yet to be fully understood. To explore its correlations, we utilized the LinkedOmics database to screen genes that are both positively and negatively associated with ALDOA in GC (Fig. 2A-B). The volcano plots indicated that ENO1 is among the top positively associated genes (Fig. 2C). Furthermore, Kyoto Encyclopedia of Genes and Genomes (KEGG) analysis showed that genes related to ALDOA are significantly enriched in glycolysis pathways (Fig. 2D). Additionally, Gene Set Enrichment Analysis (GSEA) of the previously screened ALDOA-related genes revealed a strong correlation between ALDOA and glycolysis (Fig. 2E). Gene Ontology (GO) enrichment analysis of these genes also highlighted associations with cell metabolism, suggesting a potential role for ALDOA as an oncogene that regulates cell metabolism (Fig. 2F-H).

### The correlation between ALDOA and ENO1 in GC

**Figure 3:**
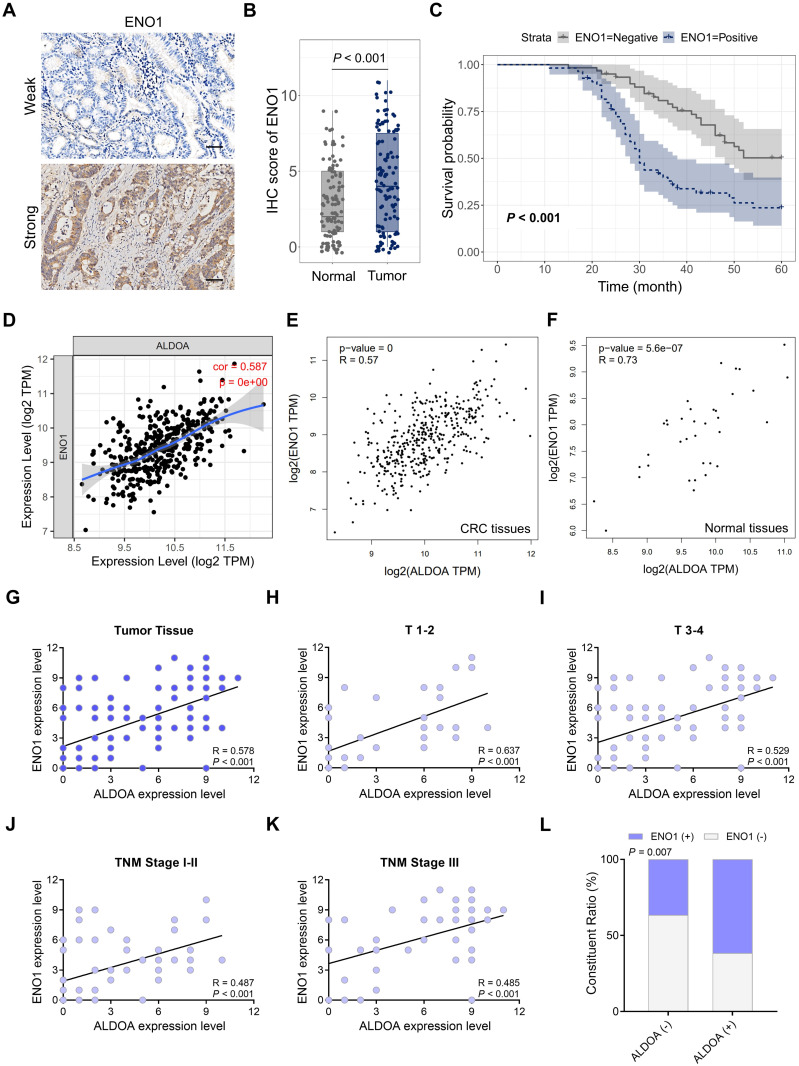
Correlation between ALDOA and ENO1 in GC tissues. (A) ENO1 expression detected by IHC in 115 GC tissues and paired normal tissues. (B) IHC score of ENO1 in (B) GC tissues and paired normal tissues. (C) Kaplan‒Meier analysis of ENO1 positive vs ENO1 negative GC patients. (D) Association between *ALDOA and ENO1* expression in GC tissues analyzed via the LinkedOmics database. (E-F) Association between *ALDOA/ENO1* expression in (E) GC tissues and (F) normal tissues analyzed via the GEPIA database. (G) Association between ALDOA and ENO1 expression according to the IHC score in GC tissues. (H-K) Association between ALDOA and ENO1 expression according to IHC score in (H) the GC with T1-2, (I) the GC with T3-4, (J) the GC with TNM I-II, and (K) the GC with TNM III. (L) Constituent ratio showing the correlation between ALDOA and ENO1 expression in GC. Scale bar = 100 μm.

The activation of the ENO1 signaling pathway has been linked to glycolysis and drug resistance in various cancers 14-16. Consequently, we evaluated ENO1 expression in 115 GC tissues and their corresponding normal tissues using IHC (Fig. 3A). Our findings revealed that ENO1 expression was significantly higher in GC tissues compared to normal tissues (Fig. 3B). Patients with ENO1-positive GC tissues exhibited a poor prognosis (*P* < 0.001, Fig. 3C). To further investigate the relationship between *ALDOA* and *ENO1* expression in GC tissues, we utilized the LinkedOmics database and the GEPIA database. A positive correlation between* ALDOA* and *ENO1* was observed (*P* < 0.001, Fig. 3D-E). Additionally, this positive correlation was also present in normal tissues (*P* < 0.001, Fig. 3F).

To illustrate these findings, we performed a linear analysis of ALDOA and ENO1 expression levels in GC tissues using the IHC score, revealing a significant positive correlation between the two (*P* < 0.001, Fig. 3G). Additionally, subgroup analyses demonstrated that within both the T1-2 and T3-4 subgroups, ALDOA expression exhibited a strong correlation with ENO1 expression in GC tissues (*P* < 0.001, Fig. 3H-I). Similarly, in the TNM stage I-II or III subgroup, there was a notable positive relationship between ALDOA and ENO1 expression (*P* < 0.001, Fig. 3J-K). Furthermore, the IHC staining results for ALDOA and ENO1 were categorized into negative and positive groups, showing a positive correlation in the ratio of ALDOA and ENO1 expression in GC (Fig. 3L).

### ALDOA affects the infiltration of immune cells in GC

**Figure 4:**
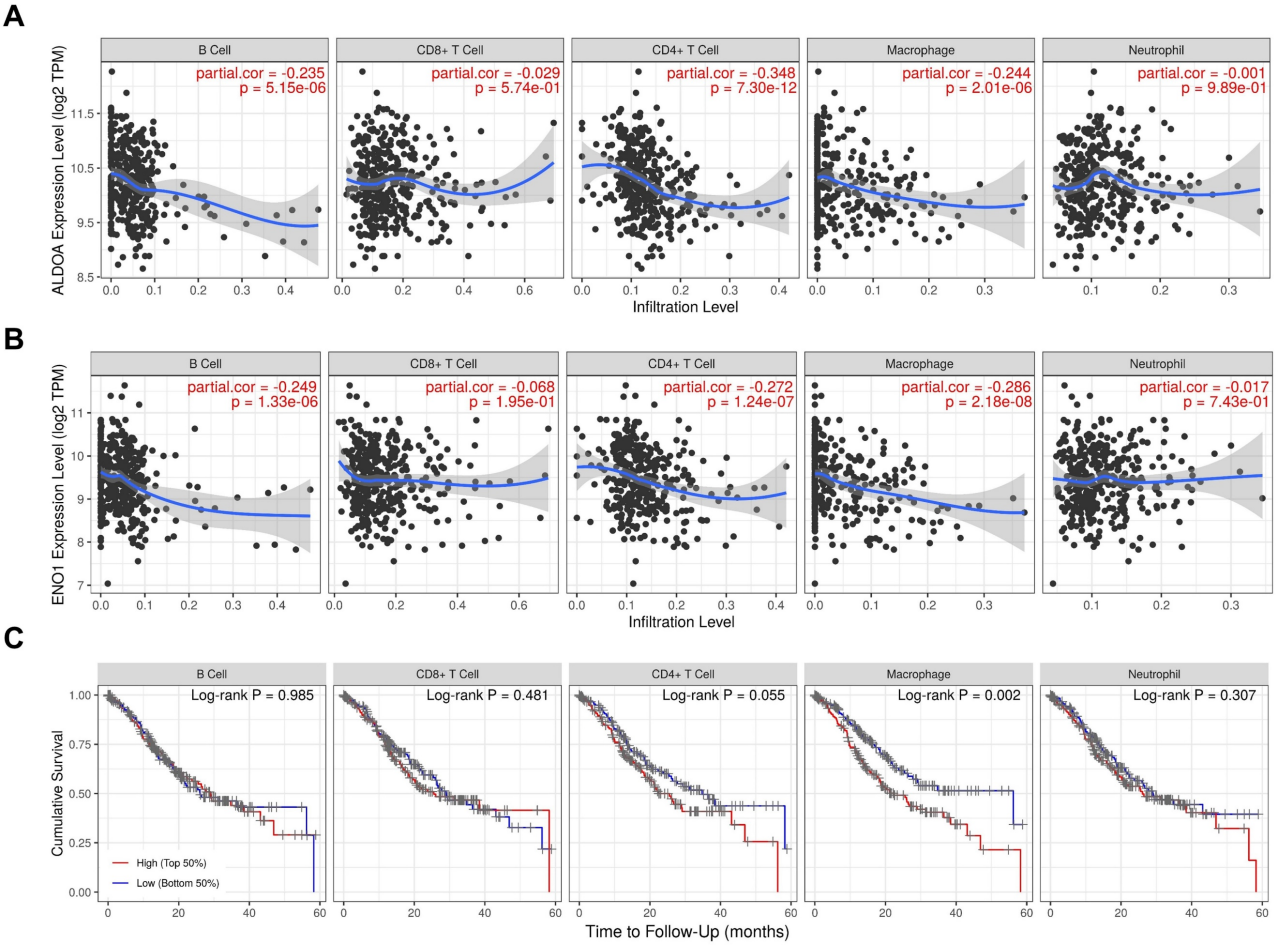
Bioinformatics analysis predicting the relationship between *ALDOA/ENO1* and infiltrating immune cells in GC. (A) The correlation between *ALDOA* expression and the infiltration of immune cells in GC according to the TIMER database. (B) The correlation between *ENO1* expression and immune cell infiltration in GC according to the TIMER database. (C) The associations between infiltrating immune cells and overall survival in GC patients were assessed using the TIMER database.

Tumor metabolic reprogramming often occurs alongside the activation of tumor-associated immune cells. Through bioinformatics analysis, we predicted the relationship between ALDOA/ENO1 and infiltrating immune cells in GC. The TIMER results confirmed that in GC, ALDOA is associated with B cells, CD4+ T cells, and macrophages in the infiltrating tumor tissues (*P* < 0.001, Fig. 4A). Interestingly, ENO1 is also related to B cells, CD4+ T cells, and macrophages in the infiltrating tumor tissues (*P* < 0.001, Fig. 4B). Therefore, our findings suggest that ALDOA influences the progression of GC by regulating immune cell infiltration, presenting a promising avenue for future research. Survival analysis revealed that high levels of macrophages in infiltrating tumor tissues impact the survival of GC patients (*P* = 0.002, Fig. 4C). This suggests that ALDOA/ENO1 may influence the survival of GC patients by affecting the infiltration of tumor-associated macrophages (TAMs).

### The influence of ALDOA/ENO1 overexpression on prognosis in GC patients

**Figure 5:**
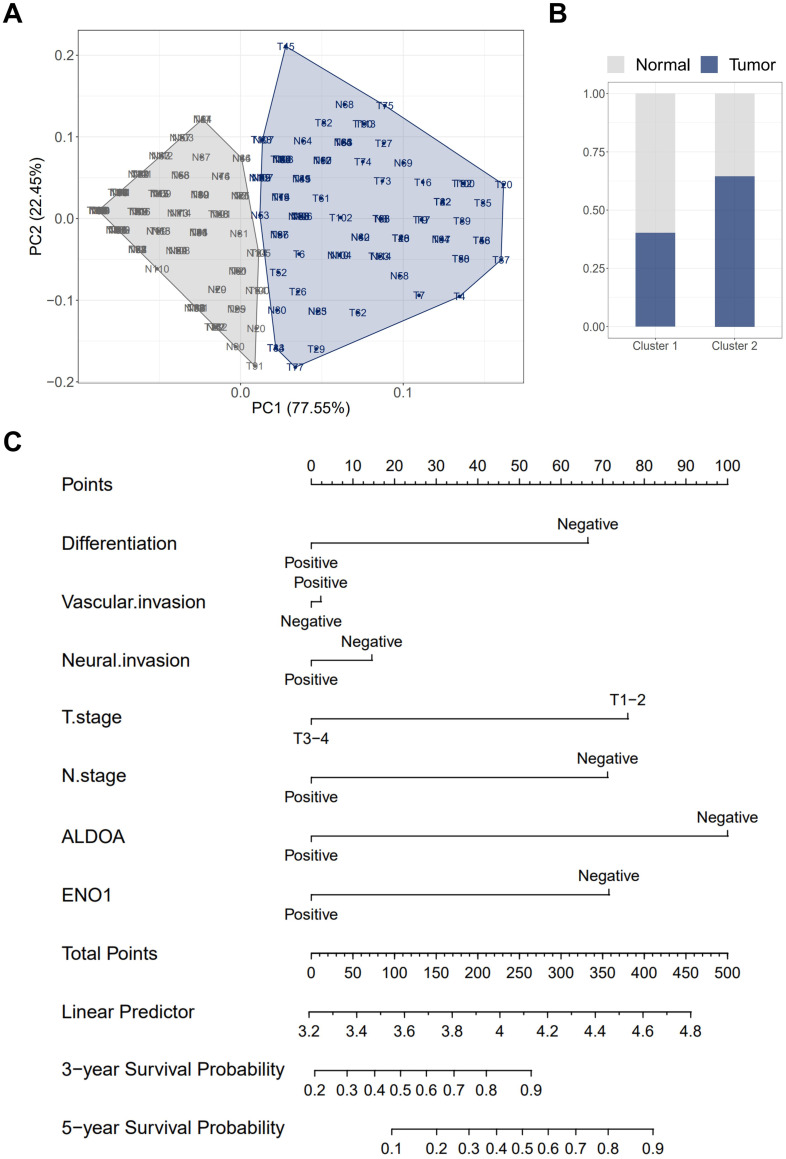
The role of ALDOA expression in GC tissues was investigated by cluster analysis and nomograms. (A) The stratification of GC and paired normal tissues in Cluster 1 and Cluster 2 based on the ALDOA/ENO1 IHC score. (B) The percentage of GC and paired normal tissues in each cluster. (C) The 3- and 5-year overall survival rates were predicted by the total points, which were calculated by each prognostic factor.

Due to the unusually high expression of ALDOA and ENO1 in gastric cancer (GC) tissues, we sought to differentiate GC tissues from normal tissues based on their expression levels. However, cluster analysis showed that 40.1% of tumor tissues and 59.9% of normal tissues were grouped into Cluster 1, while 64.5% of tumor tissues and 35.5% of normal tissues were placed in Cluster 2 (Fig. 5A-B). The results of the cluster analysis indicate that the expression levels of ALDOA and ENO1 are insufficient to accurately distinguish GC tissues from normal tissues. This could be due to the fact that the expression of ALDOA and ENO1 is closely linked in both GC tissues and normal tissues.

Subsequently, we conducted a Cox proportional hazards analysis. The univariate analysis revealed that several factors, including the degree of tumor differentiation, vascular and neural invasion, depth of tumor infiltration, lymph node metastasis, as well as the expression levels of ALDOA and ENO1, significantly influenced patient survival (*P* < 0.05, Table 1). Multivariate analysis further identified ALDOA and ENO1 expression as independent prognostic indicators. These results underscore the critical role of ALDOA and ENO1 in gastric cancer (GC) patient outcomes. Consequently, we developed a nomogram based on the Cox proportional hazards model to predict survival. The predicted survival rates were derived from the cumulative scores assigned to each prognostic factor on the nomogram scale. Crucially, the expression levels of ALDOA and ENO1 were pivotal in forecasting the 3- and 5-year overall survival of GC patients (Fig. 5C).

## Discussion

GC is one of the most prevalent malignant tumors in the digestive tract 17. Despite the fact that advancements in surgical techniques and emerging treatment modalities like immunotherapy have enhanced the prognosis of numerous tumors, the treatment options for GC remain restricted, and many patients with advanced GC have a poor survival rate 1, 2. Consequently, the identification of new therapeutic targets and the exploration of the underlying potential mechanisms are of utmost importance for the treatment of GC.

Metabolic reprogramming facilitates tumor cell growth through increasing ATP production, supplying precursors for macromolecule synthesis, and decreasing the production of reactive oxygen species (ROS) within cancer cells 18, 19. Certain oncogenes boost glycolysis and encourage cancer cell proliferation by elevating the expression of specific glucose transporters and glycolytic enzymes 20-22. ALDOA has an essential function in glycolysis 8, 23. In this study, we examined ALDOA expression in 115 GC tissues and corresponding normal tissues via IHC, and discovered that ALDOA expression was significantly increased in GC tissues, and high ALDOA levels were indicative of a poor prognosis.

We screened genes that are positively and negatively correlated with ALDOA in GC using the LinkedOmics database. The outcomes demonstrated that the expression of ALDOA was associated with glycolysis. Volcano plots indicated that ENO1 was among the top positively associated genes. Subsequently, we evaluated the correlation between ALDOA and ENO1 in GC. Additionally, we examined ENO1 expression in 115 GC tissues and paired normal tissues through IHC. We found that ENO1 expression in GC tissues was significantly higher than that in normal tissues, and patients with GC tissues showing positive ENO1 expression had a poor prognosis. According to both the LinkedOmics database and the GEPIA database, there was a positive correlation between ALDOA and ENO1 in both tumor and normal tissues. The proliferation and migration of tumor cells necessitate continuous energy, during which ALDOA and ENO1 might be abnormally activated in cancer cells 9, 24. Mechanistically, this co-expression pattern suggests a potential therapeutic strategy: targeting ALDOA/ENO1-dependent glycolysis could disrupt tumor metabolic reprogramming.

Tumor metabolic reprogramming is usually accompanied by the activation of tumor-associated immune cells 25-28. We predicted the relationship between ALDOA/ENO1 and infiltrating immune cells in GC via bioinformatics analysis. This finding implies that ALDOA/ENO1 may impact the survival of GC patients by influencing the infiltration of TAMs. Translating these findings to GC, combinatorial strategies targeting ALDOA/ENO1 with immunotherapy may synergize by reversing macrophage-mediated immunosuppression, as ALDOA/ENO1 expression correlates with tumor-associated macrophage infiltration.

We carried out a cluster analysis based on the expression of ALDOA/ENO1 in tumor and normal tissues. It was unable to effectively differentiate tumor tissue from normal tissue, which could be due to the strong correlation between their expression in both normal and tumor tissues. Then, we performed Cox proportional hazard analysis to screen the relevant factors affecting the survival of GC patients and constructed a survival-related nomogram. The expression of ALDOA and ENO1 significantly influenced the survival prognosis of GC patients. Notably, this study introduces a novel survival prediction nomogram integrating ALDOA and ENO1 expression. This nomogram may enable personalized risk stratification for GC patients, assisting adjuvant therapy decisions.

Despite the valuable insights this study provides regarding the prognostic significance and association between ALDOA and ENO1 in gastric cancer, it has several limitations. The sample size of gastric cancer patients is relatively small. A larger sample would enhance the statistical power, allowing for more accurate subgroup analyses and better generalization of the findings. This small sample size might also affect the reliability of the survival prediction nomogram, potentially leading to less precise prognostic predictions. In addition, the study is retrospective, which inherently brings selection bias. The data were collected from patients who had already undergone radical gastrectomy, and unaccounted factors in this selection process could have influenced the results. Finally, while the bioinformatics and IHC analysis identified associations between ALDOA, ENO1, glycolysis, and immune cell infiltration, the study lacks functional experiments. In further research, we will incorporate functional studies to better understand the underlying mechanisms and validate the potential of ALDOA and ENO1 as therapeutic targets.

In conclusion, it was observed that the expression levels of ALDOA and ENO1 in GC tissues were significantly elevated compared to those in adjacent normal tissues. Moreover, the abnormal expression of ALDOA/ENO1 was associated with an unfavorable prognosis in GC patients. Bioinformatics analysis indicated that ALDOA and ENO1 are involved in glycolysis and exhibit a positive correlation in their expression. The survival prediction nomogram, derived from the univariate analysis of Cox proportional hazards model data, demonstrated that the expression of ALDOA and ENO1 had a pronounced impact on the prognosis of GC patients. This finding might offer a novel approach for the combined targeted therapy of GC patients.

## Supplementary Material

Supplementary information: clinical parameters.

## Figures and Tables

**Table 1:** Results of univariate and multivariate analyses of postoperative patient survival by the Cox proportional hazard model.

Variables	Univariate analysis			Multivariate analysis		
	HR	95% CI	*P*	HR	95% CI	*P*
Age (≤60 or >60 years)	0.913	0.563-1.479	0.711			
Gender (Male/Female)	1.349	0.777-2.345	0.288			
Size of tumor (≤5 or >5 cm)	0.718	0.424-1.214	0.216			
Degree of differentiation (moderate-well/poor)	0.521	0.292-0.929	0.027^a^	0.599	0.331-1.084	0.090
Vascular invasion (negative/positive)	0.424	0.260-0.691	0.001^b^	0.883	0.495-1.578	0.675
Neural invasion (negative/positive)	0.433	0.262-0.716	0.001^b^	0.616	0.337-1.126	0.116
Depth of tumor invasion (T1-2/T3-4)	0.360	0.201-0.645	0.001^b^	0.688	0.342-1.383	0.294
Lymph node metastasis (negative/positive)	0.265	0.146-0.482	<0.001^c^	0.495	0.245-1.001	0.050
ALDOA expression (negative/positive)	0.314	0.191-0.517	<0.001^c^	0.380	0.225-0.642	<0.001^c^
ENO1 expression (negative/positive)	0.382	0.232-0.628	<0.001^c^	0.574	0.339-0.971	0.039^ a^

^a^ *P* < 0.05, ^b^ *P* < 0.01, ^c^ *P* < 0.001
