# Supplementary material for: Prognostic significance and potential association between ALDOA and ENO1 in gastric cancer
Source: J Cancer. 2025 Aug 22;16(13):3874–83. doi: 10.7150/jca.114369 (PMC12490978; doi:10.7150/jca.114369)
Supplement: Supplementary file 1 — Supplementary information: clinical parameters. [file jcav16p3874s1.pdf]

Number	Gender	Age	Neural invasion	Vascular invasion	Size of tumor	Degree of differentiation	Depth of tumor invasion	Lymph node metastasis	TNM stage	Overall survival	Status	Tumor-ALDOA	Normal-ALDOA	Tumor-ENO1	Normal-ENO1
1	Male	<65	Negative	Negative	<5cm	Poor	T1-2	Negative	I-II	60	0	Negative	Negative	Negative	Negative
2	Male	<65	Positive	Negative	<5cm	Poor	T3-4	Positive	III	24	1	Negative	Negative	Positive	Negative
3	Male	<65	Negative	Positive	>=5cm	Poor	T1-2	Positive	I-II	38	1	Negative	Positive	Positive	Positive
4	Male	>=65	Positive	Positive	<5cm	Poor	T3-4	Positive	III	25	1	Positive	Negative	Positive	Negative
5	Female	<65	Positive	Negative	<5cm	Poor	T3-4	Negative	I-II	48	0	Negative	Negative	Negative	Negative
6	Female	<65	Positive	Negative	<5cm	Poor	T3-4	Negative	I-II	29	1	Negative	Negative	Positive	Negative
7	Male	>=65	Positive	Positive	<5cm	Poor	T3-4	Positive	III	31	1	Positive	Negative	Positive	Negative
8	Female	>=65	Negative	Negative	<5cm	Poor	T1-2	Negative	I-II	47	0	Negative	Negative	Negative	Negative
9	Male	<65	Positive	Negative	<5cm	Poor	T3-4	Positive	III	34	0	Positive	Positive	Positive	Positive
10	Male	<65	Positive	Positive	<5cm	Poor	T3-4	Positive	I-II	33	0	Negative	Positive	Negative	Positive
11	Male	<65	Negative	Negative	<5cm	Moderate-well	T3-4	Positive	III	31	1	Negative	Negative	Negative	Negative
12	Male	>=65	Negative	Positive	<5cm	Moderate-well	T1-2	Positive	I-II	48	1	Positive	Negative	Negative	Positive
13	Female	>=65	Negative	Negative	<5cm	Moderate-well	T1-2	Negative	I-II	60	0	Negative	Negative	Negative	Negative
14	Female	<65	Negative	Negative	>=5cm	Poor	T3-4	Positive	I-II	29	1	Negative	Positive	Negative	Negative
15	Male	<65	Positive	Negative	<5cm	Poor	T3-4	Positive	I-II	28	1	Positive	Negative	Negative	Negative
16	Male	<65	Positive	Positive	<5cm	Poor	T3-4	Positive	III	18	1	Positive	Negative	Positive	Negative
17	Male	>=65	Positive	Positive	>=5cm	Poor	T3-4	Positive	III	26	1	Positive	Negative	Positive	Negative
18	Male	<65	Negative	Positive	>=5cm	Moderate-well	T1-2	Positive	I-II	22	1	Positive	Negative	Positive	Negative
19	Female	>=65	Negative	Positive	>=5cm	Moderate-well	T1-2	Negative	I-II	60	0	Negative	Negative	Negative	Negative
20	Female	>=65	Positive	Positive	<5cm	Poor	T3-4	Positive	III	27	1	Positive	Negative	Positive	Positive
21	Female	<65	Negative	Positive	<5cm	Poor	T3-4	Positive	III	60	0	Negative	Positive	Positive	Negative
22	Male	<65	Positive	Positive	<5cm	Poor	T3-4	Positive	III	17	1	Positive	Negative	Positive	Negative
23	Male	<65	Positive	Negative	<5cm	Poor	T3-4	Negative	I-II	38	0	Positive	Negative	Positive	Positive
24	Male	>=65	Negative	Positive	>=5cm	Poor	T3-4	Positive	I-II	33	1	Negative	Negative	Negative	Negative
25	Male	>=65	Negative	Negative	>=5cm	Poor	T3-4	Negative	I-II	45	0	Negative	Negative	Positive	Negative
26	Male	>=65	Negative	Negative	<5cm	Poor	T1-2	Positive	I-II	50	1	Negative	Negative	Positive	Negative
27	Female	<65	Positive	Negative	<5cm	Poor	T3-4	Positive	III	11	1	Positive	Negative	Positive	Positive
28	Male	>=65	Negative	Negative	<5cm	Poor	T1-2	Negative	I-II	60	0	Negative	Negative	Negative	Negative
29	Male	<65	Positive	Negative	<5cm	Poor	T3-4	Negative	I-II	30	1	Negative	Negative	Positive	Positive
30	Male	>=65	Negative	Positive	<5cm	Poor	T1-2	Positive	I-II	43	1	Negative	Negative	Negative	Positive
31	Female	<65	Negative	Negative	<5cm	Moderate-well	T1-2	Negative	I-II	60	0	Positive	Negative	Negative	Negative
32	Male	<65	Positive	Positive	>=5cm	Poor	T3-4	Positive	III	22	1	Positive	Negative	Positive	Negative
33	Female	>=65	Positive	Negative	<5cm	Moderate-well	T3-4	Positive	III	34	1	Negative	Positive	Positive	Positive
34	Male	>=65	Positive	Positive	>=5cm	Moderate-well	T3-4	Positive	III	22	1	Negative	Negative	Positive	Negative
35	Male	>=65	Positive	Negative	<5cm	Moderate-well	T3-4	Positive	III	24	0	Positive	Negative	Positive	Positive
36	Male	>=65	Negative	Negative	<5cm	Poor	T1-2	Negative	I-II	51	1	Negative	Positive	Negative	Negative
37	Female	>=65	Positive	Positive	<5cm	Poor	T1-2	Positive	III	19	0	Positive	Negative	Positive	Negative
38	Female	<65	Positive	Positive	<5cm	Poor	T3-4	Positive	III	25	1	Positive	Negative	Positive	Negative
39	Male	<65	Negative	Negative	<5cm	Moderate-well	T3-4	Positive	III	60	0	Negative	Negative	Positive	Negative
40	Male	>=65	Positive	Positive	<5cm	Poor	T3-4	Positive	III	27	1	Positive	Positive	Positive	Positive
41	Female	>=65	Negative	Negative	<5cm	Poor	T1-2	Negative	I-II	60	0	Negative	Negative	Negative	Negative
42	Female	<65	Positive	Positive	<5cm	Moderate-well	T3-4	Positive	I-II	37	1	Positive	Negative	Positive	Positive
43	Male	<65	Positive	Positive	<5cm	Poor	T3-4	Positive	I-II	34	1	Positive	Negative	Negative	Negative
44	Male	<65	Negative	Negative	<5cm	Poor	T1-2	Positive	I-II	29	1	Negative	Positive	Positive	Negative
45	Male	>=65	Positive	Positive	<5cm	Poor	T3-4	Positive	III	28	1	Positive	Positive	Negative	Negative
46	Male	>=65	Negative	Positive	<5cm	Poor	T3-4	Positive	I-II	23	1	Positive	Negative	Positive	Negative
47	Male	>=65	Positive	Positive	<5cm	Poor	T3-4	Positive	I-II	38	1	Positive	Positive	Negative	Positive
48	Male	>=65	Positive	Negative	>=5cm	Moderate-well	T3-4	Negative	I-II	28	1	Positive	Negative	Positive	Negative
49	Female	>=65	Positive	Positive	<5cm	Moderate-well	T3-4	Positive	I-II	32	0	Positive	Positive	Negative	Negative
50	Female	>=65	Negative	Negative	>=5cm	Moderate-well	T1-2	Positive	III	18	1	Positive	Positive	Positive	Negative
51	Male	<65	Positive	Positive	>=5cm	Moderate-well	T3-4	Positive	I-II	60	0	Negative	Negative	Positive	Negative
52	Male	>=65	Positive	Positive	>=5cm	Moderate-well	T3-4	Positive	III	54	1	Negative	Positive	Positive	Negative
53	Male	>=65	Positive	Negative	>=5cm	Moderate-well	T3-4	Negative	I-II	60	0	Positive	Negative	Negative	Negative
54	Male	<65	Positive	Positive	<5cm	Poor	T3-4	Positive	III	25	1	Positive	Positive	Negative	Negative
55	Male	<65	Negative	Negative	<5cm	Moderate-well	T1-2	Negative	I-II	60	0	Negative	Negative	Positive	Negative
56	Male	>=65	Negative	Positive	<5cm	Poor	T1-2	Positive	III	26	1	Positive	Negative	Positive	Negative
57	Female	>=65	Positive	Positive	<5cm	Poor	T3-4	Negative	I-II	22	1	Negative	Negative	Negative	Negative
58	Female	>=65	Negative	Negative	>=5cm	Poor	T3-4	Negative	I-II	15	1	Negative	Positive	Negative	Positive
59	Male	>=65	Positive	Positive	<5cm	Poor	T3-4	Positive	I-II	46	1	Negative	Negative	Negative	Negative
60	Male	<65	Positive	Positive	>=5cm	Poor	T3-4	Positive	III	36	1	Negative	Positive	Positive	Positive
61	Male	<65	Positive	Negative	<5cm	Poor	T1-2	Negative	I-II	43	0	Positive	Negative	Positive	Negative
62	Female	<65	Positive	Positive	<5cm	Poor	T3-4	Positive	III	27	1	Negative	Negative	Positive	Negative
63	Male	<65	Negative	Negative	<5cm	Poor	T1-2	Positive	I-II	60	0	Positive	Positive	Positive	Negative
64	Male	<65	Positive	Negative	<5cm	Poor	T3-4	Positive	I-II	37	0	Negative	Positive	Positive	Negative
65	Male	>=65	Negative	Positive	>=5cm	Poor	T1-2	Positive	III	25	0	Positive	Negative	Negative	Negative
66	Male	>=65	Positive	Negative	<5cm	Moderate-well	T3-4	Positive	I-II	46	1	Negative	Negative	Negative	Positive
67	Female	>=65	Positive	Positive	<5cm	Poor	T1-2	Positive	I-II	60	0	Negative	Negative	Negative	Negative
68	Female	>=65	Positive	Positive	<5cm	Poor	T3-4	Positive	III	46	1	Negative	Positive	Negative	Negative
69	Male	<65	Positive	Negative	<5cm	Poor	T3-4	Negative	I-II	37	1	Negative	Positive	Negative	Positive
70	Female	>=65	Negative	Negative	<5cm	Poor	T1-2	Negative	I-II	52	1	Positive	Negative	Negative	Positive
71	Female	<65	Positive	Negative	<5cm	Poor	T3-4	Negative	I-II	60	0	Negative	Negative	Negative	Negative
72	Male	<65	Positive	Negative	<5cm	Poor	T3-4	Positive	III	60	0	Negative	Negative	Negative	Negative
73	Male	<65	Positive	Positive	<5cm	Poor	T3-4	Positive	III	30	1	Positive	Negative	Positive	Negative
74	Male	>=65	Negative	Negative	<5cm	Moderate-well	T3-4	Negative	I-II	49	1	Positive	Negative	Positive	Negative
75	Male	>=65	Negative	Negative	<5cm	Poor	T1-2	Positive	I-II	25	0	Positive	Positive	Negative	Negative
76	Female	>=65	Negative	Negative	>=5cm	Moderate-well	T3-4	Negative	I-II	60	0	Negative	Negative	Negative	Negative
77	Male	>=65	Negative	Negative	<5cm	Poor	T3-4	Negative	I-II	28	1	Negative	Negative	Positive	Negative
78	Female	<65	Negative	Negative	<5cm	Poor	T1-2	Positive	I-II	60	0	Negative	Negative	Negative	Negative
79	Male	<65	Negative	Negative	<5cm	Moderate-well	T3-4	Negative	I-II	42	1	Positive	Negative	Negative	Negative
80	Male	<65	Negative	Negative	>=5cm	Moderate-well	T1-2	Negative	I-II	60	0	Negative	Negative	Positive	Negative
81	Male	>=65	Negative	Negative	<5cm	Moderate-well	T1-2	Negative	I-II	60	0	Negative	Negative	Negative	Negative
82	Male	<65	Negative	Positive	<5cm	Poor	T1-2	Positive	I-II	48	1	Positive	Positive	Negative	Positive
83	Female	<65	Negative	Negative	<5cm	Poor	T1-2	Negative	I-II	60	0	Negative	Negative	Negative	Negative
84	Female	<65	Negative	Negative	>=5cm	Moderate-well	T1-2	Negative	I-II	41	1	Positive	Positive	Negative	Negative
85	Male	<65	Negative	Positive	<5cm	Poor	T1-2	Negative	I-II	60	0	Negative	Negative	Negative	Negative
86	Male	<65	Negative	Positive	<5cm	Moderate-well	T1-2	Negative	I-II	60	0	Negative	Negative	Negative	Positive
87	Male	>=65	Positive	Positive	<5cm	Poor	T3-4	Negative	I-II	60	0	Negative	Negative	Negative	Negative

88	Male	<65	Negative	Positive	<5cm	Poor	T3-4	Positive	III	60	0	Positive	Positive	Positive	Negative
89	Male	<65	Negative	Negative	<5cm	Poor	T3-4	Positive	III	22	1	Positive	Negative	Positive	Negative
90	Female	<65	Positive	Negative	>=5cm	Poor	T3-4	Positive	III	21	1	Positive	Negative	Negative	Positive
91	Male	<65	Negative	Negative	<5cm	Poor	T3-4	Positive	III	35	1	Negative	Negative	Positive	Positive
92	Male	<65	Positive	Positive	>=5cm	Poor	T3-4	Positive	III	30	1	Positive	Negative	Positive	Negative
93	Male	<65	Positive	Negative	<5cm	Poor	T3-4	Positive	III	60	0	Negative	Negative	Positive	Negative
94	Male	<65	Positive	Positive	<5cm	Poor	T3-4	Positive	III	21	1	Positive	Negative	Positive	Negative
95	Male	>=65	Positive	Negative	<5cm	Poor	T3-4	Negative	I-II	36	1	Positive	Positive	Negative	Negative
96	Male	>=65	Negative	Negative	<5cm	Moderate-well	T1-2	Positive	I-II	31	1	Positive	Positive	Positive	Positive
97	Male	<65	Negative	Negative	>=5cm	Moderate-well	T3-4	Positive	I-II	60	0	Negative	Positive	Positive	Negative
98	Male	>=65	Negative	Negative	<5cm	Poor	T1-2	Negative	I-II	58	0	Negative	Negative	Negative	Negative
99	Female	>=65	Negative	Negative	<5cm	Poor	T3-4	Positive	III	39	0	Negative	Negative	Negative	Positive
100	Male	>=65	Positive	Positive	>=5cm	Poor	T3-4	Positive	III	30	1	Positive	Negative	Positive	Positive
101	Male	>=65	Positive	Positive	>=5cm	Moderate-well	T3-4	Positive	III	23	0	Negative	Negative	Negative	Negative
102	Male	>=65	Positive	Positive	>=5cm	Poor	T3-4	Positive	III	24	1	Positive	Negative	Positive	Negative
103	Male	>=65	Negative	Negative	>=5cm	Moderate-well	T3-4	Positive	I-II	60	0	Positive	Negative	Negative	Negative
104	Female	>=65	Positive	Positive	>=5cm	Poor	T3-4	Positive	III	31	1	Negative	Positive	Negative	Positive
105	Female	<65	Negative	Negative	<5cm	Poor	T1-2	Negative	I-II	60	0	Negative	Negative	Negative	Positive
106	Female	>=65	Positive	Negative	>=5cm	Poor	T3-4	Negative	I-II	43	1	Negative	Positive	Negative	Positive
107	Male	<65	Negative	Negative	<5cm	Poor	T1-2	Positive	I-II	50	1	Positive	Positive	Negative	Negative
108	Female	>=65	Negative	Negative	<5cm	Moderate-well	T1-2	Negative	I-II	60	0	Negative	Negative	Negative	Negative
109	Male	<65	Negative	Negative	<5cm	Moderate-well	T1-2	Negative	I-II	60	0	Negative	Negative	Negative	Negative
110	Male	<65	Positive	Negative	<5cm	Moderate-well	T1-2	Negative	I-II	60	0	Negative	Negative	Negative	Negative
111	Female	>=65	Negative	Negative	>=5cm	Poor	T3-4	Negative	I-II	60	0	Negative	Negative	Negative	Positive
112	Male	<65	Positive	Negative	>=5cm	Moderate-well	T3-4	Positive	I-II	42	1	Negative	Negative	Positive	Negative
113	Male	<65	Negative	Positive	<5cm	Poor	T3-4	Positive	III	52	1	Positive	Negative	Negative	Negative
114	Male	<65	Negative	Negative	<5cm	Poor	T1-2	Positive	III	20	1	Positive	Negative	Positive	Negative
115	Male	>=65	Positive	Negative	<5cm	Poor	T3-4	Negative	I-II	60	0	Negative	Negative	Negative	Negative
